# Defect-engineered Fe_3_O_4_@SnO_2_ nanozyme microneedles couple local photothermal-photodynamic therapy with redox-amplified ferroptosis

**DOI:** 10.3389/fchem.2026.1991252

**Published:** 2026-10-19

**Authors:** Wei Guo, Wenbo Wang, Zhuoran Yang, Liqiang Jing

**Affiliations:** 1 Key Laboratory of Functional Inorganic Materials Chemistry (Ministry of Education), School of Chemistry and Materials Science, International Joint Research Center for Catalytic Technology, Heilongjiang University, Harbin, China; 2 Key Laboratory of Photochemical Biomaterials and Energy Storage Materials, Heilongjiang Province and College of Chemistry and Chemical Engineering, Harbin Normal University, Harbin, China

**Keywords:** dissolving microneedle, Fe_3_O_4_@SnO_2_, ferroptosis, nanocatalytic therapy, oxygen vacancy

## Abstract

Localized nanocatalytic therapy is constrained by insufficient reactive oxygen species (ROS) output, tumor antioxidant buffering, and poor retention of therapeutic agents at the lesion. Here, we report an oxygen-deficient Fe_3_O_4_@SnO_2_ nanozyme (HFC) co-delivered with gallic acid (GA) through dissolving poly(sodium acrylate)/poly(vinyl alcohol)/poly(vinylpyrrolidone) microneedles (HFCG-MN). Hollow-structure formation and stepwise calcination increased the oxygen-vacancy signal, broadened optical absorption, and lowered interfacial charge-transfer resistance. Under 655 nm irradiation, HFC produced stable, power-dependent heating together with singlet oxygen and hydroxyl radicals, while depleting approximately half of the available glutathione within 120 min. The PAAs/PVA/PVP matrix provided mechanically robust skin penetration, rapid dissolution, and acid- and light-responsive GA release. In 4T1 cells, the combined treatment generated the strongest ROS signal, disrupted lysosomal and mitochondrial homeostasis, increased intracellular Fe^2+^ and lipid peroxidation, and caused extensive cell death. In a 4T1 tumor model, HFCG-MN plus laser irradiation increased the local tumor temperature to approximately 55 °C within 10 min and restricted the mean tumor volume to 101.2 mm^3^ on day 14, compared with 871.7 mm^3^ in the control group, corresponding to a tumor-inhibition rate of 88.4%. No evident body-weight loss or major-organ histopathology was observed. These data support a local therapeutic platform that couples defect-engineered nanozyme chemistry and microneedle delivery with photothermal/photodynamic action and redox-amplified ferroptosis-associated damage.

## 1 Introduction

Ferroptosis is an iron-dependent form of regulated cell death driven by phospholipid peroxidation (Dixon et al., 2012; Stockwell et al., 2017; Jiang et al., 2021). Its therapeutic induction requires the convergence of redox-active iron, sustained ROS generation, and insufficient antioxidant repair. This convergence is difficult to achieve in solid tumors because labile iron is tightly regulated, exogenous oxidants are rapidly quenched, and nonspecific oxidative chemistry may damage normal tissues. A useful local platform should therefore increase the intratumoral iron pool and ROS output while restricting systemic exposure. The cystine-glutamate antiporter/system xC− supplies cysteine for glutathione (GSH) synthesis, and glutathione peroxidase 4 (GPX4) uses GSH to reduce membrane lipid hydroperoxides (Friedmann Angeli et al., 2014; Yang et al., 2014; Zhang et al., 2024). Thus, ROS production alone may be insufficient unless GSH/GPX4-centered buffering is simultaneously weakened. For the present system, the most relevant design requirements are therefore iron regulation, continuous oxidant generation, and antioxidant depletion (Tang et al., 2021; Ming et al., 2024). Because Fe^2+^ accumulation and lipid peroxidation are consistent with, but do not alone prove, ferroptosis, we describe the observed response as ferroptosis-associated damage.

Nanomaterials offer a way to engineer these requirements into the therapeutic agent itself. Biologically derived nanocarriers, including extracellular vesicles, further broaden the options for tumor-directed delivery and modulation of the tumor microenvironment (Cheng X. et al., 2024). Ferroptosis-based nanomedicines can transport iron, modulate antioxidant pathways, or convert external energy into localized oxidative stress, while multifunctional and biomimetic architectures improve the coordination of these processes (Shi et al., 2023; Liang et al., 2024; Xing et al., 2024; Xu et al., 2024; Malla et al., 2025; Mohapatra et al., 2024; Sheikh et al., 2024). Within this field, chemodynamic therapy (CDT) exploits Fenton or Fenton-like reactions to transform relatively weak endogenous H_2_O_2_ into highly reactive hydroxyl radicals under tumor-microenvironment conditions (Huo et al., 2017; Tang et al., 2019; Lu et al., 2026). Strategies that couple catalytic-ion delivery with GSH depletion or that organize cascade nanozyme reactions have shown why substrate supply and antioxidant consumption are as important as nominal catalytic activity (Lin et al., 2018; Jia et al., 2025; Yu et al., 2026). More broadly, nanozymes provide robust, engineerable alternatives to natural enzymes and can combine stimulus responsiveness with peroxidase-like activity (Gao et al., 2007; Jiang et al., 2019; Wu et al., 2019; Cheng C. et al., 2024; Meng et al., 2024; Sun et al., 2024). Ferrite nanoparticles are especially relevant because Fe^2+^/Fe^3+^ cycling can support Fenton chemistry, and heat or light can further accelerate ROS production (Gao et al., 2007; Sun et al., 2024). Nevertheless, catalytic therapy alone is frequently limited by finite H_2_O_2_, slow reaction kinetics near physiological pH, and tumor antioxidant capacity.

Phototherapy can supply the missing temporal trigger. Photodynamic therapy (PDT) uses excited photosensitizers to generate cytotoxic ROS, whereas photothermal therapy (PTT) converts absorbed light into heat (Dolmans et al., 2003; Cheng et al., 2014). Nano-enabled PTT/PDT has advanced toward multimodal treatment because heat can increase membrane permeability, accelerate catalytic reactions, and sensitize cells to oxidative damage, while ROS can extend injury beyond the region of maximal heating (Fan et al., 2017; Chen et al., 2023; Wang et al., 2024; Xue et al., 2025; Zheng et al., 2025). Yet simple co-administration does not guarantee synergy: optical absorption, carrier stability, oxygen availability, charge separation, and intratumoral retention remain interdependent constraints (Zhao et al., 2021). Tumor-responsive nanocarriers that modulate hypoxia or release catalytic components have illustrated the value of linking microenvironmental chemistry to externally triggered therapy (Chen et al., 2016; Ma R. et al., 2024). These observations motivate a more integrated design in which PTT and PDT are not independent add-ons to CDT, but inputs to the same redox cascade: light initiates heating and photochemical ROS, heat accelerates reaction kinetics, and iron-mediated chemistry sustains oxidative damage after excitation.

The effectiveness of such integration depends on the electronic structure of the inorganic component. Metal-oxide photocatalysts often exhibit insufficient visible-light absorption and rapid electron–hole recombination. Oxygen-vacancy engineering can introduce sub-bandgap states, alter local coordination, increase adsorption or activation of reactants, and create catalytically active sites (Pan et al., 2013; Liu et al., 2024; Su et al., 2024). Heterojunction construction provides a complementary strategy by establishing interfacial band alignment that promotes charge separation and directs carrier migration (Wang et al., 2014). Studies of surface defects, oxygen-deficient oxides, and defect location further show that activity depends not merely on the total number of vacancies but on where they reside and how they alter charge transport (Cheng C. et al., 2024; Sun et al., 2024). For an Fe_3_O_4_/SnO_2_ heterophase, these principles suggest a useful division of labor: defect-rich SnO_2_ can broaden photoresponse and provide interfacial trapping sites, whereas mixed-valence Fe_3_O_4_ can contribute peroxidase-like and Fenton chemistry. The key hypothesis is that a hollow, vacancy-rich heterostructure will couple photon absorption, charge transfer, heat generation, and iron redox cycling more effectively than an unengineered oxide mixture.

Even a chemically effective nanocatalyst can fail if it is not retained at the lesion. Microneedles bypass the stratum corneum through micron-scale channels and can deposit therapeutic cargo directly into a confined tissue volume (Prausnitz, 2004; Kim et al., 2012). Dissolving microneedles are particularly attractive because the polymer matrix serves both as a mechanically competent insertion device and as a transient drug reservoir, eliminating retrieval of a rigid implant (McCrudden et al., 2014; Ganeson et al., 2023). Their performance, however, requires a deliberate compromise among tip sharpness, fracture resistance, hydration, cargo compatibility, and dissolution rate; polymer composition and payload loading can shift all of these properties (McCrudden et al., 2014). Microneedle-assisted delivery has already demonstrated that local placement can improve the activity of biologics and combination treatments in tumor models (Cheng et al., 2023; Ma J. et al., 2024; Singh et al., 2024; Yin et al., 2025). For a photoactive nanozyme, local insertion offers an additional advantage: it spatially aligns the catalytic payload with the irradiation field, potentially reducing off-target exposure while maintaining a high intratumoral concentration. Thus, the microneedle is not merely a packaging component; it is part of the mechanism that converts nanoscale reactivity into tissue-level selectivity.

Co-loading a small phenolic molecule creates another opportunity to tune this redox network. Gallic acid (GA) has reported anticancer, antioxidant, and context-dependent pro-oxidant activities, and its biological effects vary with concentration, metal availability, and cellular redox state (Verma et al., 2013; Badhani et al., 2015). This duality makes GA potentially useful in an iron-containing, ROS-producing platform, but it also argues against assigning a single molecular function without direct pathway measurements. In the present study, GA is therefore treated as a combination payload whose contribution is assessed experimentally through release behavior and treatment response; the specific extent to which it chelates iron, perturbs GSH metabolism, or directly promotes cell death remains a testable mechanistic question.

On this basis, we designed a spatially confined, defect-regulated redox system rather than a collection of parallel treatment modalities. Hollow FeSnO(OH)_5_ precursors were etched and stepwise calcined to obtain oxygen-deficient Fe_3_O_4_@SnO_2_ nanoparticles (HFC), which were co-embedded with GA in poly(sodium acrylate) (PAAs)/poly(vinyl alcohol) (PVA)/poly (vinylpyrrolidone) (PVP) dissolving microneedles (HFCG-MN) (Scheme 1). PAAs and PVA provide the structural matrix, whereas hydrophilic PVP serves as a rapidly dissolving co-matrix component that improves formulation homogeneity and facilitates payload release. The study tests a sequential structure-delivery-mechanism hypothesis: hollowing and vacancy formation enhance light harvesting and interfacial charge transfer; microneedle insertion localizes HFC and coordinates stimulus-responsive cargo release; and 655 nm irradiation initiates heat and ROS production that are amplified by Fe^2+^/Fe^3+^ cycling and GSH consumption. The expected biological consequence is not restricted to one death pathway: oxidative pressure should drive lipid peroxidation and ferroptosis-associated damage while lysosomal leakage and mitochondrial depolarization connect the same redox cascade to apoptotic injury. By evaluating this logic from materials characterization through 4T1 cellular responses and local tumor therapy, we aim to establish how defect engineering and microneedle delivery jointly convert a photoactive nanozyme into a tumor-confined redox amplifier.

**SCHEME 1:**
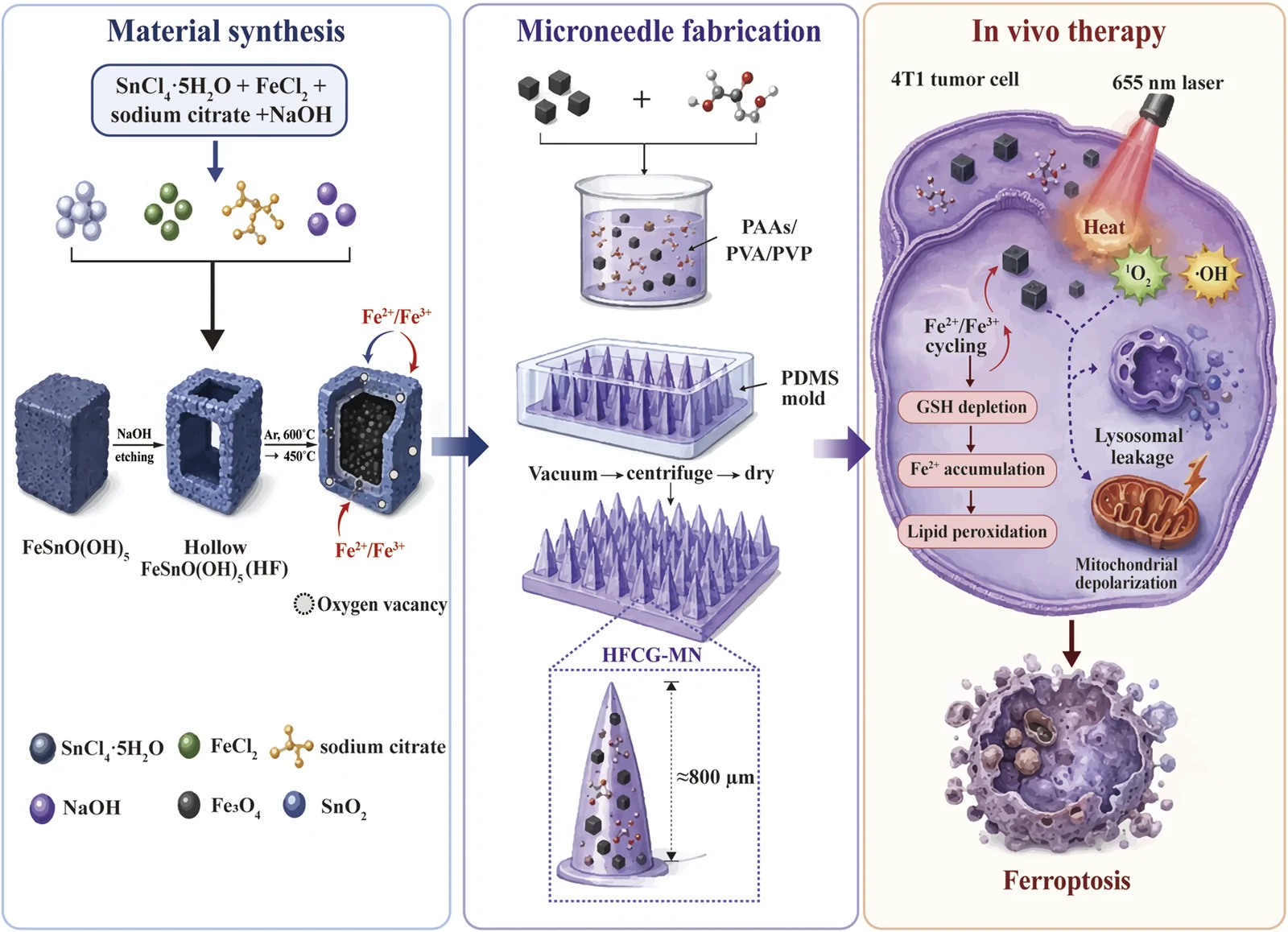
Schematic illustration of material synthesis, HFCG-loaded microneedle (HFCG-MN) fabrication, and the proposed antitumor mechanism.

## 2 Materials and methods

### 2.1 Synthesis of hollow FeSnO(OH)_5_ and HFC nanoparticles

SnCl_4_·5H_2_O (2 mmol) was dissolved in 10 mL of anhydrous ethanol and sonicated for 10 min. Sodium citrate dihydrate (2 mmol) and FeCl_2_ (2 mmol) were dissolved in 70 mL of deionized water, followed by slow addition of the tin-containing solution and stirring for 20 min. NaOH (10 mL, 2 mol L^-1^) was added dropwise and the mixture was stirred for 1 h. The precipitate was collected at 11,000 rpm for 5 min, washed three times with water and ethanol, and dried at 60 °C. To form hollow particles, 0.125 g of FeSnO(OH)_5_ was dispersed in 10 mL of water, mixed with 10 mL of NaOH (8 mol L^-1^), stirred for 2 h, washed, and vacuum-dried at 60 °C to obtain hollow FeSnO(OH)_5_ (HF). For the two-step treatment, HF was placed in a tube furnace under continuously flowing Ar, heated to 600 °C at 2.5 °C min^-1^, and held for 2 h. Without removing the sample or cooling it to room temperature, the furnace was then cooled to 450 °C under Ar and held at 450 °C for another 2 h. The furnace was finally allowed to cool to room temperature under Ar, yielding HFC.

### 2.2 Fabrication of HFCG-MN

PAAs (0.1 g) and PVA (1.0 g) were mixed in a water bath at 80 °C for 2 h. PVP solution (2 mL, 1 g mL^-1^) was then added and stirred for 30 min. In the final formulation, PAAs and PVA constituted the mechanically supportive matrix, whereas hydrophilic PVP acted as a rapidly dissolving co-matrix component that improved precursor homogeneity and facilitated dissolution and payload release. After cooling, HFC (0.02 g mL^-1^) and GA (0.01 g mL^-1^) were incorporated. The mixture was loaded into a polydimethylsiloxane microneedle mold, vacuum-treated for 10 min, centrifuged at 2,000 rpm for 10 min, refilled, degassed, dried under vacuum at 37 °C, and demolded. The final array contained 10 × 10 needles. Mechanical strength was assessed using repeated aluminum-foil puncture and force-displacement testing at 0.08 mm min^-1^. Dissolution was visualized in agar using methylene blue (MB), and skin microchannel closure was monitored after insertion.

### 2.3 Physicochemical, photothermal, and catalytic characterization

Morphology and elemental distribution were analyzed by scanning electron microscopy (SEM), transmission electron microscopy (TEM), and energy-dispersive X-ray spectroscopy (EDS) mapping. Phase and surface chemistry were evaluated by X-ray diffraction (XRD) and X-ray photoelectron spectroscopy (XPS); oxygen vacancies were assessed by electron spin resonance (ESR). Optical absorption and interfacial charge-transfer behavior were examined by ultraviolet-visible (UV-vis) diffuse reflectance spectroscopy and electrochemical impedance spectroscopy (EIS), respectively. HFC dispersions (0.25–1.0 mg mL^-1^) or HFCG-MN were irradiated at 655 nm. Power-dependent experiments used 0.5, 1.0, and 2.0 W cm^-2^; unless otherwise specified, comparative photothermal and ROS experiments were performed at 1.0 W cm^-2^. Temperature was recorded with infrared thermography, and five laser on-off cycles were used to evaluate photothermal stability. Singlet oxygen was measured by 1,3-diphenylisobenzofuran (DPBF) bleaching at 420 nm and 2,2,6,6-tetramethylpiperidine (TEMP)-trapped ESR. Hydroxyl-radical formation was evaluated using 3,3′,5,5′-tetramethylbenzidine oxidation, MB degradation, and 5,5-dimethyl-1-pyrroline N-oxide (DMPO)-trapped ESR across irradiation times, laser powers, HFC concentrations, and pH values. For GSH depletion, 2 mL of nanoparticle dispersion (0.5 mg mL^-1^) was mixed with 1 mL GSH (5 mmol L^-1^) at 37 °C. At 0–120 min, residual thiol content was detected with 5,5′-dithiobis (2-nitrobenzoic acid) at 412 nm.

### 2.4 GA release and cytocompatibility

HFCG-MN was immersed in buffer at pH 7.4, representing the normal physiological extracellular environment, or at pH 5.4, representing a strongly acidified condition relevant to intracellular endosomal/lysosomal compartments and providing a stringent condition for evaluating acid-responsive release. The samples were subjected to alternating 5 min laser-on and 10 min laser-off periods using a 655 nm laser at 1.0 W cm^-2^. GA release was quantified at 270 nm using a calibration curve. For cytocompatibility, L929 fibroblasts and 4T1 cells were incubated with HFC, GA, or microneedle-matrix components for 24 h. 3-(4,5-dimethylthiazol-2-yl)-2,5-diphenyltetrazolium bromide (MTT) reagent was added for 4 h, and absorbance was recorded at 490 nm.

### 2.5 Cellular oxidative damage and ferroptosis-associated readouts

4T1 cells were assigned to control (C), laser alone (L), HFC, HFC plus laser (HFC + L), or HFC co-formulated with GA (HFCG) plus laser (HFCG + L) groups. For all laser-treated cellular groups, irradiation was performed at 655 nm and 1.0 W cm^-2^. Live/dead status was assessed by calcein acetoxymethyl ester/propidium iodide (Calcein-AM/PI) staining. Intracellular ROS, lysosomal integrity, Fe^2+^, lipid peroxidation (LPO), and mitochondrial membrane potential were evaluated using 2′,7′-dichlorodihydrofluorescein diacetate (DCFH-DA), acridine orange (AO), FerroOrange, BODIPY 581/591 C11, and JC-1, respectively.

### 2.6 *In vivo* local tumor therapy

Female BALB/c mice (4–6 weeks old) bearing subcutaneous 4T1 tumors of approximately 100 mm^3^ were randomized to C, L, HFC-loaded microneedles (HFC-MN), HFC-MN + L, or HFCG-MN + L groups. Microneedles were pressed onto the tumor surface, and the irradiated groups received 655 nm laser exposure at 1.0 W cm^-2^ for approximately 10 min. Tumor surface temperature was monitored by infrared thermography. Body weight and tumor dimensions were recorded during 14 days of treatment; tumor volume was calculated as length × width^2^/2. At endpoint, tumors and major organs were collected for gross analysis and hematoxylin and eosin (H&E) staining.

## 3 Results and discussion

### 3.1 Synthesis and structural characterization of defect-engineered HFC

FeSnO(OH)_5_ precursors were first synthesized via a coprecipitation method, and the effects of the iron source and its dosage on the morphology of the products were systematically investigated. SEM images showed that the use of other iron sources resulted in irregular morphologies and severe aggregation, whereas FeCl_2_ yielded uniformly dispersed particles (Supplementary Figures S1a–c). The amount of FeCl_2_ also played a critical role in controlling the product morphology. An insufficient amount of the iron precursor led to incomplete reaction, whereas excess FeCl_2_ caused pronounced aggregation and morphological deformation (Supplementary Figures S1d–f). Among the investigated conditions, 2 mmol of FeCl_2_ produced well-defined cubic particles with uniform dimensions, intact morphology, and negligible aggregation. Therefore, 2 mmol of FeCl_2_ was selected as the optimal condition for the synthesis of FeSnO(OH)_5_. The HF structure was subsequently obtained through NaOH etching, accompanied by the formation of oxygen vacancies (Supplementary Figure S2). TEM images revealed a distinct hollow interior while retaining a regular block-like morphology. Elemental mapping further demonstrated the homogeneous distribution of Fe, Sn, and O throughout the particles, indicating that the etching process preserved the compositional uniformity of the precursor (Supplementary Figure S3). The XRD pattern of HF matched well with the standard diffraction pattern of FeSnO(OH)_5_ (JCPDS No. 31–0654), without detectable impurity peaks, confirming its high phase purity (Supplementary Figure S4a). Particle-size analysis showed an average particle size of approximately 120 nm, demonstrating good uniformity of the as-prepared HF particles (Supplementary Figure S4b). To optimize the thermal treatment, HF was first calcined at 300, 450, 600, and 750 °C, and the corresponding morphological evolution was examined by SEM (Supplementary Figure S5). At relatively low temperatures, the particles largely retained the block-like morphology of the precursor, with only slight surface shrinkage caused by dehydration. With increasing temperature, nanoscale pores gradually developed on the particle surfaces. However, excessive calcination resulted in severe sintering and the formation of dense, large aggregates. These results demonstrate that the calcination temperature strongly influences the structural evolution and particle dispersion of HF. A two-step calcination strategy was subsequently investigated using different temperature combinations, including 600 + 300 °C, 600 + 450 °C, 600 + 600 °C, and 450 + 450 °C. Significant differences in both morphology and phase composition were observed among the resulting samples. In particular, the sample obtained by sequential calcination at 600 °C and 450 °C exhibited the most favorable particle dispersion and an appropriate degree of interparticle aggregation. This condition avoided the excessive aggregation of fine particles observed at lower thermal gradients while suppressing severe sintering and structural densification induced by harsher thermal treatment. Moreover, characteristic diffraction peaks corresponding to Fe_3_O_4_ were detected, confirming the coexistence of Fe^2+^ and Fe^3+^ species. Such mixed-valence Fe centers provide redox-active sites that are favorable for the subsequent catalytic performance of the material. Therefore, the 600 + 450 °C two-step calcination protocol was selected as the optimal thermal treatment condition (Supplementary Figure S6). TEM images of the optimized HFC sample revealed a well-defined block-like morphology with a clearly discernible hollow interior (Figure 1a). ESR spectra showed characteristic oxygen-vacancy signals at g = 2.003 for FeSnO(OH)_5_, HF, and HFC. Notably, HFC exhibited a substantially stronger ESR signal than the other two samples, demonstrating a pronounced increase in oxygen-vacancy concentration after etching and calcination (Figure 1b). EDS elemental mapping further confirmed the homogeneous distribution of Fe, Sn, and O throughout HFC, indicating that the multicomponent composition remained spatially uniform during the structural reconstruction process (Figure 1c).

**FIGURE 1:**
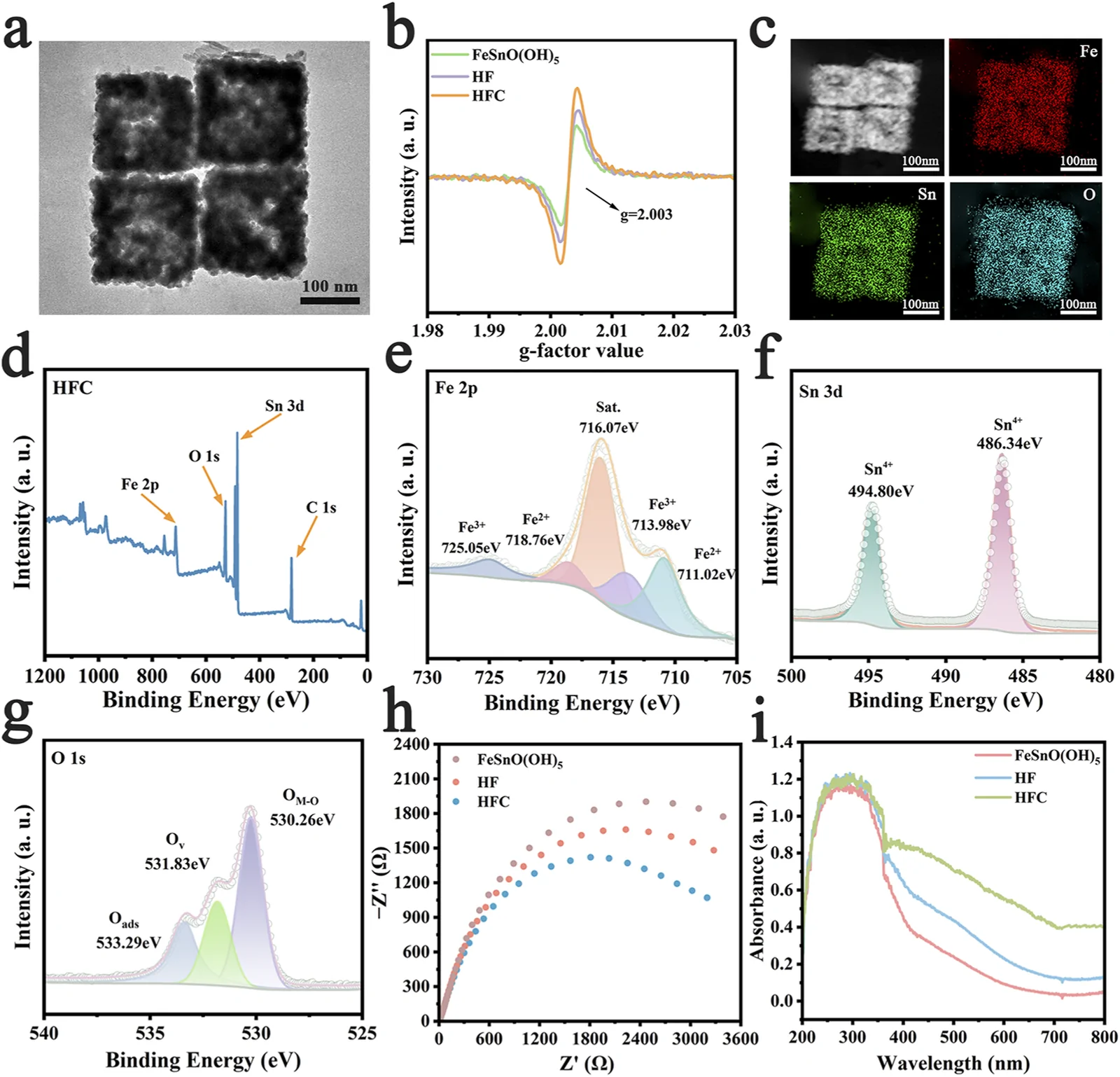
Structural and electronic characterization of defect-engineered HFC. **(a)** TEM image of hollow HFC. **(b)** Oxygen-vacancy ESR spectra of FeSnO(OH)_5_, HF, and HFC. **(c)** High-angle annular dark-field scanning transmission electron microscopy image and corresponding Fe, Sn, and O elemental maps. **(d)** XPS survey spectrum. High-resolution **(e)** Fe 2p, **(f)** Sn 3d, and **(g)** O 1s spectra. **(h)** EIS spectra of FeSnO(OH)_5_, HF, and HFC and **(i)** UV-vis diffuse-reflectance spectra.

The surface chemical states of HFC were further investigated by XPS (Figures 1d–g). In the Fe 2p spectrum, the peaks at 725.05 and 718.76 eV were assigned to Fe^3+^ and Fe^2+^ species in the Fe 2p_1/2_ region, respectively, while the peaks at 713.98 and 711.02 eV were attributed to Fe^3+^ and Fe^2+^ species in the Fe 2p_3/2_ region. The peak at 716.07 eV was assigned to the Fe 2p satellite signal (Sui et al., 2021; Yang et al., 2023). The characteristic peaks at 494.80 and 486.34 eV were attributed to the Sn 3d_3/2_ and Sn 3d_5/2_ states of Sn^4+^, respectively (Ma et al., 2020). The O 1s spectrum could be deconvoluted into three components centered at 533.29, 531.83, and 530.26 eV, corresponding to surface-adsorbed hydroxyl species, oxygen-deficient sites, and metal–oxygen bonds, respectively (Shen et al., 2025). Collectively, these results confirm the successful construction of a hollow, oxygen-vacancy-rich HFC structure containing mixed-valence Fe species. Electrochemical impedance spectroscopy (EIS) was further employed to evaluate the interfacial charge-transfer behavior of the samples (Figure 1h). Generally, a smaller semicircle diameter in the Nyquist plot corresponds to a lower charge-transfer resistance. HFC exhibited the smallest semicircle among the tested samples, indicating the lowest interfacial charge-transfer resistance and more efficient electron transport. The improved charge-transfer kinetics are beneficial for carrier migration and utilization, thereby facilitating subsequent ROS generation. The optical absorption properties of FeSnO(OH)_5_, HF, and HFC were investigated by UV–vis diffuse reflectance spectroscopy (Figure 1i). Compared with FeSnO(OH)_5_ and HF, HFC exhibited markedly enhanced absorption over the visible and near-infrared regions. This enhanced light-harvesting capability can be attributed to the combined effects of its hollow architecture and abundant oxygen vacancies, which are favorable for photothermal conversion.

### 3.2 Photothermal performance and oxidative stress amplification of HFC

To evaluate the photothermal performance of HFC, we first examined concentration-dependent heating under 655 nm irradiation (1.0 W cm^-2^). After 10 min, the temperature of the 1.0 mg mL^-1^ HFC dispersion reached approximately 62 °C, whereas lower concentrations produced smaller temperature increases (Figure 2a). The heating and natural-cooling data were fitted to a standard photothermal-conversion model (Figure 2b), yielding a system time constant of 194.25 s and a photothermal conversion efficiency of approximately 46% (R^2^ = 0.997). HFC also exhibited nearly unchanged heating and cooling profiles over five consecutive irradiation cycles, indicating good photothermal stability (Figure 2c). The power-dependent photothermal behavior of HFC was further evaluated under 655 nm irradiation. When the laser power density increased from 0.5 to 2.0 W cm^-2^, both the heating rate and final temperature increased markedly, confirming the efficient and controllable photothermal response of HFC (Supplementary Figure S7). The final temperatures at 0.5, 1.0, and 2.0 W cm^-2^ were 39.1, 55.0, and 66.2 °C, respectively. We next investigated the ability of HFC to promote ROS generation. Singlet oxygen (^1^O_2_) production was monitored using 1,3-diphenylisobenzofuran (DPBF). Under laser irradiation, HFC induced a substantially faster decrease in the characteristic absorption of DPBF than HFC-1, HF, and FeSnO(OH)_5_ (Figures 2d,e). The C/C_0_ value continuously decreased with increasing HFC concentration or laser power density (Figure 2f), and TEMP-trapped ESR showed the strongest characteristic 1:1:1 triplet signal for HFC (Figure 2g). The ·OH-generating activity was further assessed using TMB as a chromogenic probe. Oxidation of TMB produces blue oxTMB with a characteristic absorption peak at 652 nm. The absorbance at 652 nm increased progressively with reaction time, and HFC produced a substantially stronger response than HFC-1, HF, and FeSnO(OH)_5_, demonstrating its superior ·OH-generating activity (Supplementary Figure S8). Increasing the laser power density from 0.5 to 2.0 W cm^-2^ further enhanced TMB oxidation (Supplementary Figure S9). In addition, the oxTMB signal increased with HFC concentration and was consistently higher at pH 5.4 than at pH 7.4, confirming that the Fenton-like catalytic activity of HFC was concentration-dependent and favored by acidic conditions (Supplementary Figure S10). Consistent with the TMB results, DMPO-trapped ESR showed a much stronger characteristic 1:2:2:1 signal for HFC than for FeSnO(OH)_5_, HF, and HFC-1 (Figure 2h), and the signal intensity progressively increased with irradiation time (Figure 2i). HFC also consumed approximately 50% of GSH within 120 min and showed stronger depletion under acidic conditions (Supplementary Figure S11). Together, the photothermal, chromogenic-probe, ESR, and GSH-depletion results demonstrate that HFC establishes a light-enhanced oxidative-stress amplification process.

**FIGURE 2:**
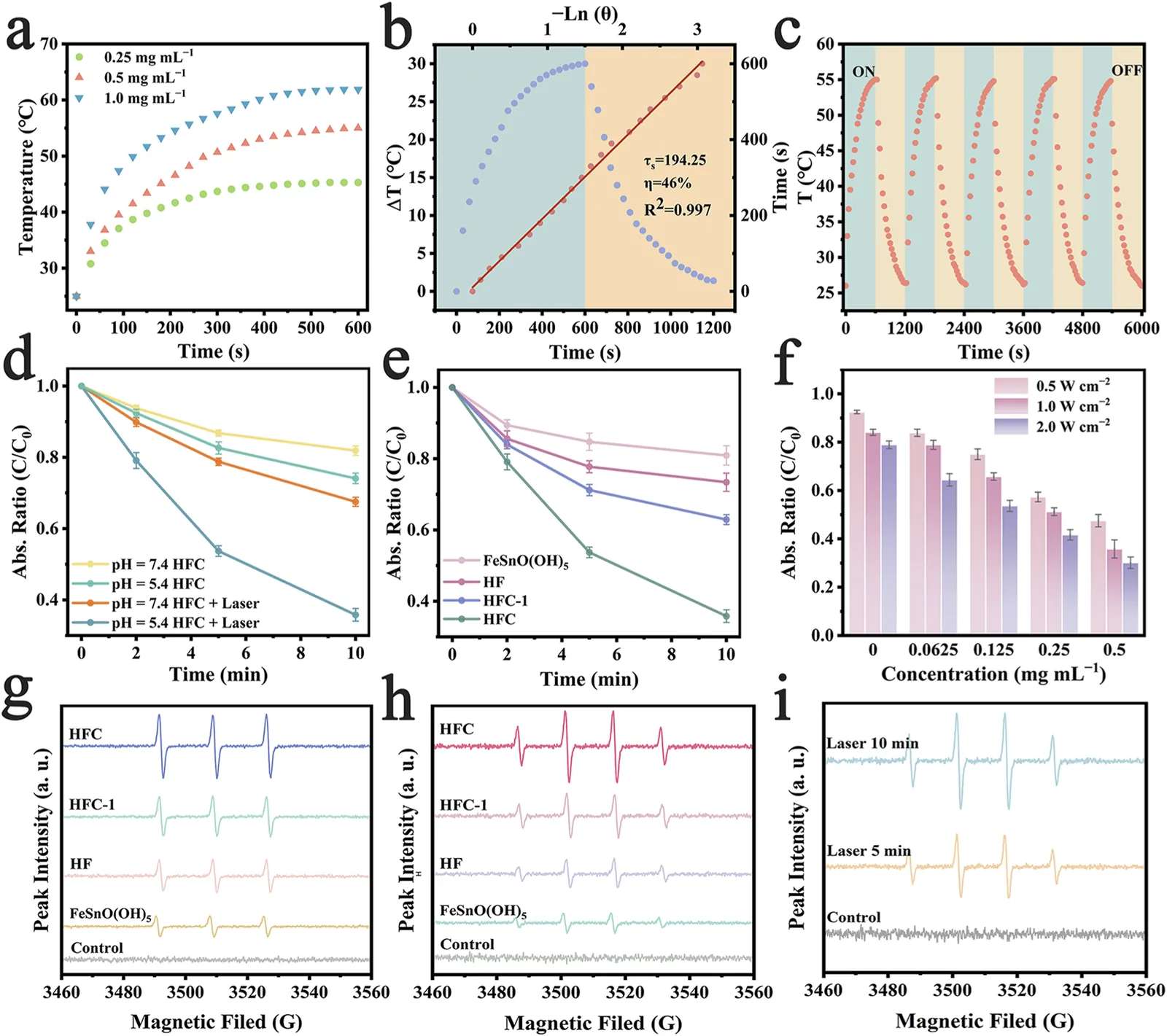
Light-triggered photothermal and ROS-generating performance of HFC. **(a)** Concentration-dependent temperature changes of HFC dispersions under 655 nm irradiation at 1.0 W cm^-2^. **(b)** Photothermal-conversion fitting based on the heating and natural-cooling profile, used to determine the system time constant and photothermal conversion efficiency. **(c)** Five laser on-off cycles at 1.0 W cm^-2^. DPBF-based singlet-oxygen measurements under **(d)** pH/irradiation conditions, **(e)** different material groups, and **(f)** different HFC concentrations and laser powers. **(g)** TEMP-trapped ESR spectra. DMPO-trapped ESR spectra for **(h)** different materials and **(i)** different irradiation times.

### 3.3 Fabrication and functional characterization of HFCG-MN

The microneedle formulation was first optimized by evaluating the effects of PAAs and PVA contents on microneedle morphology and mechanical performance. Microneedles fabricated from 0.05 g of PAAs underwent pronounced deformation during molding or demolding, resulting in poorly defined structures. Increasing the amount of PAAs to 0.2 g markedly increased the viscosity of the matrix, leading to severe aggregation and adhesion of the microneedle tips, blurred tip structures, and irregular arrays, all of which are unfavorable for effective skin penetration. In contrast, microneedles prepared with 0.1 g of PAAs exhibited well-defined tips and a uniform array without obvious structural defects (Supplementary Figure S12). Therefore, 0.1 g of PAAs was selected for subsequent formulation optimization, and PVA was further incorporated to improve the mechanical strength of the microneedles. The effect of PVA content on microneedle formation and mechanical properties was subsequently investigated. When the PVA content was 0.5 g, the improvement in mechanical strength was limited and the microneedles remained mechanically insufficient. Although increasing the PVA content to 1.5 g substantially enhanced the mechanical strength, the excessive viscosity of the precursor matrix impaired demolding and resulted in pronounced deformation and void formation, leading to poor structural integrity (Supplementary Figure S13). By comparison, microneedles containing 1.0 g of PVA exhibited the best overall performance, with sharp tips, compact and well-organized arrays, and no obvious adhesion or defects. Foil penetration tests further confirmed their sufficient mechanical strength. These results indicate that the combination of 0.1 g of PAAs and 1.0 g of PVA provide a favorable balance between mechanical strength and molding quality. PVP was subsequently incorporated as a hydrophilic, rapidly dissolving co-matrix component to improve formulation homogeneity and facilitate payload release; thus, the final HFCG-MN polymer matrix consisted of PAAs/PVA/PVP. SEM observations further showed that the fabricated microneedles formed a uniform and well-organized 10 × 10 array. The tip-to-tip spacing, microneedle height, and maximum base diameter were approximately 650, 800, and 300 μm, respectively (Figures 3a–c). Elemental mapping revealed homogeneous distributions of O, Sn, and Fe throughout the microneedle matrix, confirming the uniform dispersion of HFC within the microneedles (Figures 3d–f). The mechanical durability of HFCG-MN was further evaluated by repeated penetration through 10 μm-thick foil. HFCG-MN maintained effective penetration performance, demonstrating good structural robustness (Figure 3g). Consistently, mechanical testing showed that PAAs + PVA-MN exhibited a higher load-bearing capacity than PAAs-MN, demonstrating that PVA incorporation effectively reinforced the PAAs-based microneedle matrix (Figure 3h). GA was subsequently incorporated into the microneedles as a therapeutic payload, and the photothermal properties of HFCG-MN were evaluated. The temperature increase of HFCG-MN showed a clear dependence on laser power density, and infrared thermal images showed a corresponding increase in surface temperature with increasing laser power, demonstrating controllable photothermal heating (Figures 3i,j). In addition, the heating and cooling profiles remained nearly unchanged over repeated laser on–off cycles, indicating good photothermal cycling stability (Figure 3k). To visualize the swelling and dissolution behavior of the microneedles, MB-loaded microneedles were inserted into agar gel as a tissue-mimicking model. Under laser irradiation, the blue signal surrounding the microneedle tips gradually intensified with time, reflecting the progressive release and diffusion of MB. After approximately 120 s, the microneedle tips were almost completely dissolved, demonstrating rapid laser-assisted dissolution and payload release (Supplementary Figure S14a). The dissolution process of HFCG-MN was further monitored by removing the microneedle patches from agar at predetermined time intervals. Under laser irradiation, the microneedle array was almost completely dissolved within approximately 240 s, with little visible residue, further demonstrating the rapid dissolution behavior of HFCG-MN (Supplementary Figure S14b). The release behavior of GA from HFCG-MN was then quantitatively investigated. UV–vis absorption spectra of GA solutions at different concentrations were first recorded to establish a calibration curve. A strong linear relationship between GA concentration and absorbance was obtained, providing a reliable basis for quantitative analysis of GA release (Figures 3l,m). As shown in Figure 3n, GA release from the microneedles displayed both pH-dependent and laser-regulated behavior. Compared with neutral conditions, the cumulative release of GA was markedly enhanced under acidic conditions. Laser irradiation further accelerated GA release, whereas the release rate decreased after the laser was switched off. These results demonstrate the acid-responsive and light-regulated release behavior of HFCG-MN. In this experiment, pH 7.4 represented the normal physiological extracellular environment, whereas pH 5.4 represented a strongly acidified condition relevant to intracellular endosomal/lysosomal compartments rather than the typical bulk extracellular pH of solid tumors.

**FIGURE 3:**
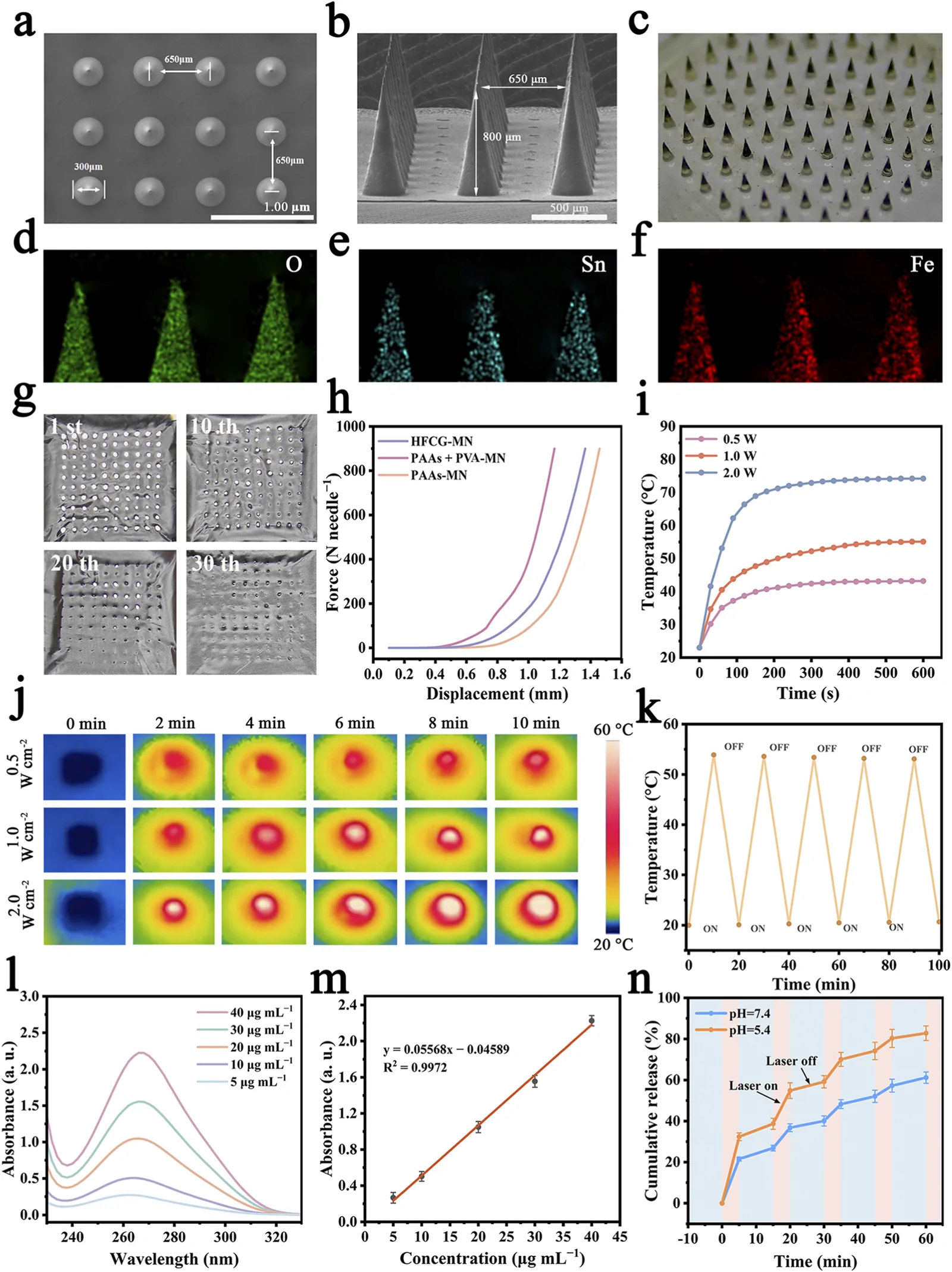
SEM images of HFCG-MN from **(a)** top and **(b)** side views. **(c)** Photograph of HFCG-MN. **(d–f)** Elemental mapping images of O, Sn, and Fe in HFCG-MN. **(g)** Repeated penetration performance of HFCG-MN through 10 μm-thick foil. **(h)** Mechanical properties of the indicated microneedle formulations. **(i)** Photothermal heating curves of HFCG-MN under 655 nm laser irradiation at 0.5, 1.0, and 2.0 W cm^-2^. **(j)** Corresponding infrared thermal images. **(k)** Photothermal cycling stability of HFCG-MN under repeated 655 nm laser on–off irradiation at 1.0 W cm^-2^. **(l)** UV–vis absorption spectra of GA solutions at different concentrations. **(m)** Calibration curve of GA based on the characteristic absorbance. **(n)** GA release profiles from HFCG-MN at pH 5.4 and 7.4 under alternating 655 nm irradiation (1.0 W cm^-2^).

### 3.4 *In vitro* antitumor activity of HFCG-MN

The cytocompatibility of HFC, GA, and the microneedle matrix materials was first evaluated using the MTT assay in normal L929 fibroblasts and 4T1 tumor cells. At the highest tested HFC concentration of 500 μg mL^-1^, the cell viability remained approximately 96% in L929 cells but decreased to 78% in 4T1 cells at the highest tested concentration, indicating good compatibility with normal cells together with a moderate inhibitory effect on tumor cells (Figure 4a). In comparison, even at a GA concentration of 500 μg mL^−1^, the viabilities of L929 and 4T1 cells remained approximately 97% and 90%, respectively, demonstrating low cytotoxicity of free GA toward normal cells but only limited antitumor activity when used alone (Figure 4b). These results indicate that GA alone is insufficient to achieve efficient tumor cell killing under the tested conditions and support its incorporation into a combined therapeutic system. Moreover, the different microneedle matrix materials, including PAAs, PVA, and PAAs + PVA + PVP, showed negligible cytotoxicity toward both L929 and 4T1 cells, confirming their good cytocompatibility as carriers for subsequent therapeutic applications (Figure 4c). Because excessive intracellular ROS accumulation plays an important role in oxidative damage and tumor cell death, intracellular ROS levels in 4T1 cells after different treatments were evaluated using the DCFH-DA probe (Figures 4d,e). The control and laser groups exhibited only weak green fluorescence, indicating low basal ROS levels. A modest increase in fluorescence was observed after HFC treatment, whereas HFC + L produced substantially stronger green fluorescence, confirming enhanced ROS generation upon laser irradiation. Notably, the HFCG + L group exhibited the strongest fluorescence signal, demonstrating that incorporation of GA further intensified intracellular oxidative stress under the combined treatment. To investigate whether ferroptosis was involved in HFCG-mediated tumor cell killing, intracellular Fe^2+^ levels and LPO were subsequently examined. FerroOrange staining showed weak fluorescence in the control and laser groups, whereas HFC treatment markedly increased the intracellular Fe^2+^ signal. An even stronger fluorescence intensity was observed in the HFC + L and HFCG + L groups, indicating a substantial increase in the intracellular labile Fe^2+^ pool after the combined treatments (Figure 4g; Supplementary Figure S15). The integrity of lysosomes was further evaluated using AO staining. Compared with the control groups, HFCG + L treatment caused a pronounced change in the AO fluorescence pattern, with enhanced green fluorescence, consistent with lysosomal membrane destabilization and impaired lysosomal integrity (Figure 4f). Mitochondrial membrane potential was further assessed using the JC-1 probe. The control and laser groups displayed predominantly strong red fluorescence, indicating well-maintained mitochondrial membrane potential. HFC treatment caused a slight reduction in red fluorescence, whereas HFC + L resulted in a pronounced increase in green fluorescence.

**FIGURE 4:**
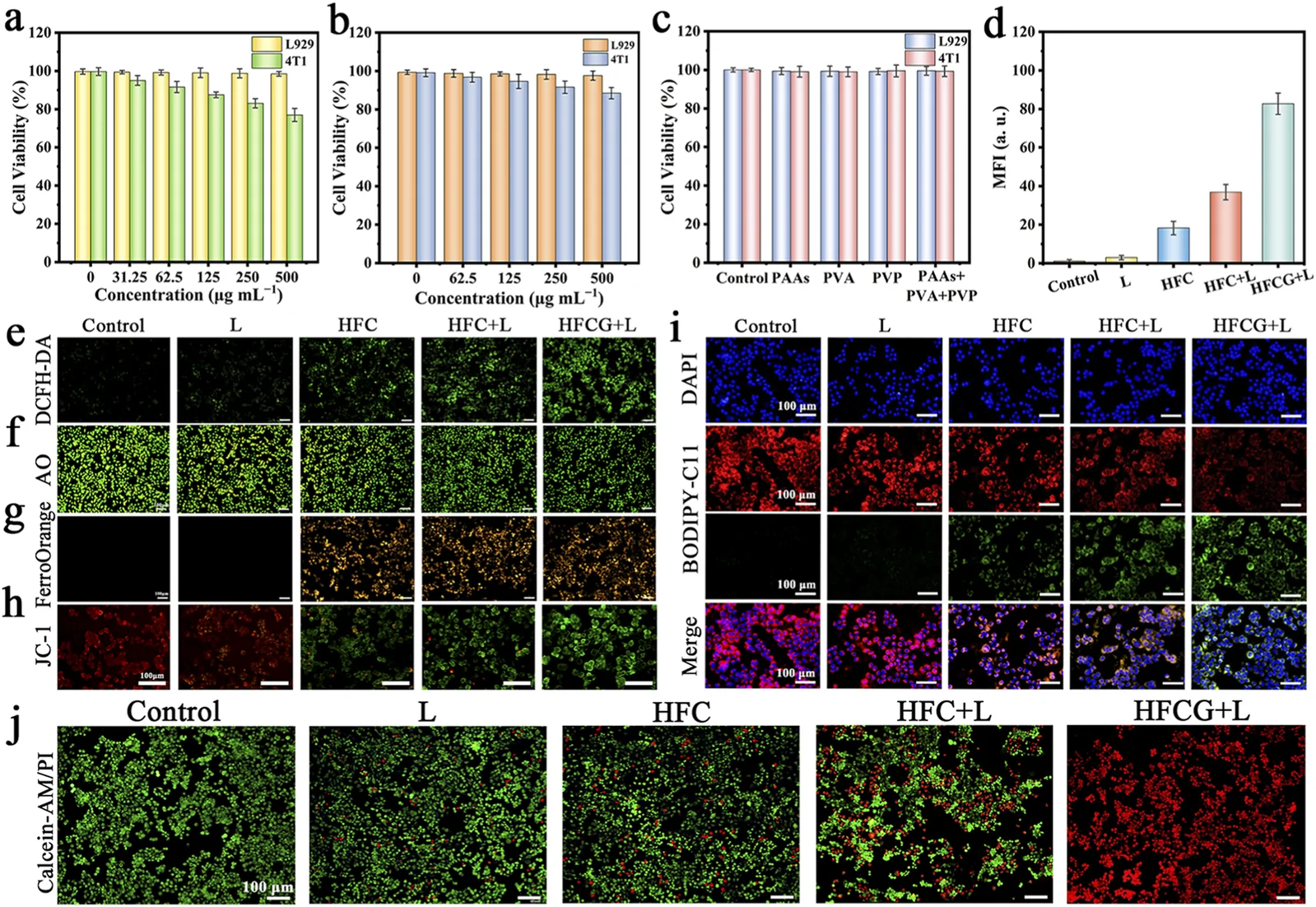
Cell viabilities of L929 and 4T1 cells treated with different concentrations of **(a)** HFC and **(b)** GA. **(c)** Cell viabilities of L929 and 4T1 cells treated with different microneedle matrix materials (PAAs, PVA, and PAAs + PVA + PVP). Intracellular ROS **(d)** quantitative analysis and **(e)** corresponding fluorescence images in 4T1 cells using DCFH-DA. **(f)** AO fluorescence images for evaluating lysosomal integrity. **(g)** Intracellular Fe^2+^ fluorescence images detected using FerroOrange. **(h)** JC-1 fluorescence images for evaluating mitochondrial membrane potential. **(i)** Lipid peroxidation detected using BODIPY 581/591 C11. **(j)** Live/dead cell staining using Calcein-AM/PI.

The HFCG + L group showed the most obvious red-to-green fluorescence transition, indicating severe mitochondrial membrane depolarization and mitochondrial dysfunction under the combined treatment (Figure 4h; Supplementary Figure S16). LPO, a key biochemical feature associated with ferroptosis, was further evaluated using the BODIPY 581/591 C11 probe. The control and laser groups were dominated by red fluorescence with little green fluorescence, indicating minimal lipid oxidation. In contrast, green fluorescence progressively increased in the HFC and HFC + L groups and reached the highest level in the HFCG + L group, demonstrating extensive accumulation of lipid peroxides (Figure 4i). Together with the increased intracellular Fe^2+^ level and enhanced ROS generation, these results support the involvement of ferroptosis-associated oxidative damage in HFCG + L-mediated tumor cell killing. The elevated Fe^2+^ pool can facilitate Fenton-like reactions, thereby amplifying ROS production and lipid peroxidation and ultimately aggravating oxidative damage in tumor cells. Finally, Calcein-AM/PI staining was performed to directly visualize the viability of 4T1 cells after different treatments (Figure 4j; Supplementary Figure S17). The control and laser groups exhibited predominantly green fluorescence with almost no red fluorescence, indicating high cell viability. HFC treatment resulted in a small increase in PI-positive red fluorescence, whereas HFC + L markedly increased the proportion of dead cells. Notably, the HFCG + L group showed extensive red fluorescence accompanied by a substantial loss of green fluorescence, demonstrating the strongest tumor cell-killing effect among all groups. These results further confirm that the combination of HFCG with laser irradiation significantly enhances tumor cell death through intensified oxidative stress, mitochondrial dysfunction, and ferroptosis-associated lipid peroxidation.

### 3.5 *In vivo* antitumor efficacy and biosafety of HFCG-MN

To evaluate the *in vivo* therapeutic efficacy of HFCG-MN, its antitumor performance was systematically investigated in 4T1 tumor-bearing mice. The mice were randomly divided into five groups: (1) control, (2) laser (L), (3) HFC-MN, (4) HFC-MN + L, and (5) HFCG-MN + L. After establishment of the 4T1 tumor model, the indicated treatments were initiated on day 1, followed by continuous monitoring of tumor growth and therapeutic outcomes (Figure 5a). The recovery of skin microchannels after HFCG-MN insertion was first examined to assess the reversibility of microneedle-induced skin disruption. Immediately after insertion, a clear microneedle array pattern with regularly distributed microchannels was observed on the skin surface. The microchannel marks gradually faded over time and were almost completely closed within 20 min, with no obvious skin damage, redness, or swelling (Figure 5b). These results demonstrate that HFCG-MN can efficiently create transient microchannels for transdermal delivery while allowing rapid restoration of the skin barrier after microneedle removal, indicating good skin compatibility of the microneedle system. The *in vivo* photothermal performance of HFCG-MN under laser irradiation was subsequently evaluated using infrared thermal imaging. As shown in Figure 5c, the temperature of the tumor region in the laser-only group increased only slightly during 10 min of irradiation, indicating a weak photothermal effect. In contrast, the tumor temperature in both the HFC-MN + L and HFCG-MN + L groups increased rapidly with irradiation time and reached approximately 55 °C after 10 min. Notably, the high-temperature region was mainly confined to the tumor site, with limited heat diffusion to the surrounding tissue. These results confirm the strong and localized photothermal response of the microneedle system, which provides favorable conditions for effective photothermal tumor ablation. The therapeutic efficacy was further evaluated by continuously monitoring tumor growth. As shown by the tumor growth curves in Figure 5d, tumors in the control, laser, and HFC-MN groups grew rapidly throughout the treatment period, whereas tumor growth was markedly inhibited in the HFC-MN + L group. Among all treatments, HFCG-MN + L produced the strongest tumor suppression. Consistently, the average tumor weight measured on day 14 was significantly lower in the HFCG-MN + L group than in the other groups, in agreement with the tumor volume results (Figure 5e). In addition, no obvious decrease in body weight was observed in any treatment group during the 14-day experimental period, indicating that the treatment did not cause detectable systemic toxicity based on body weight monitoring (Figure 5f).

**FIGURE 5:**
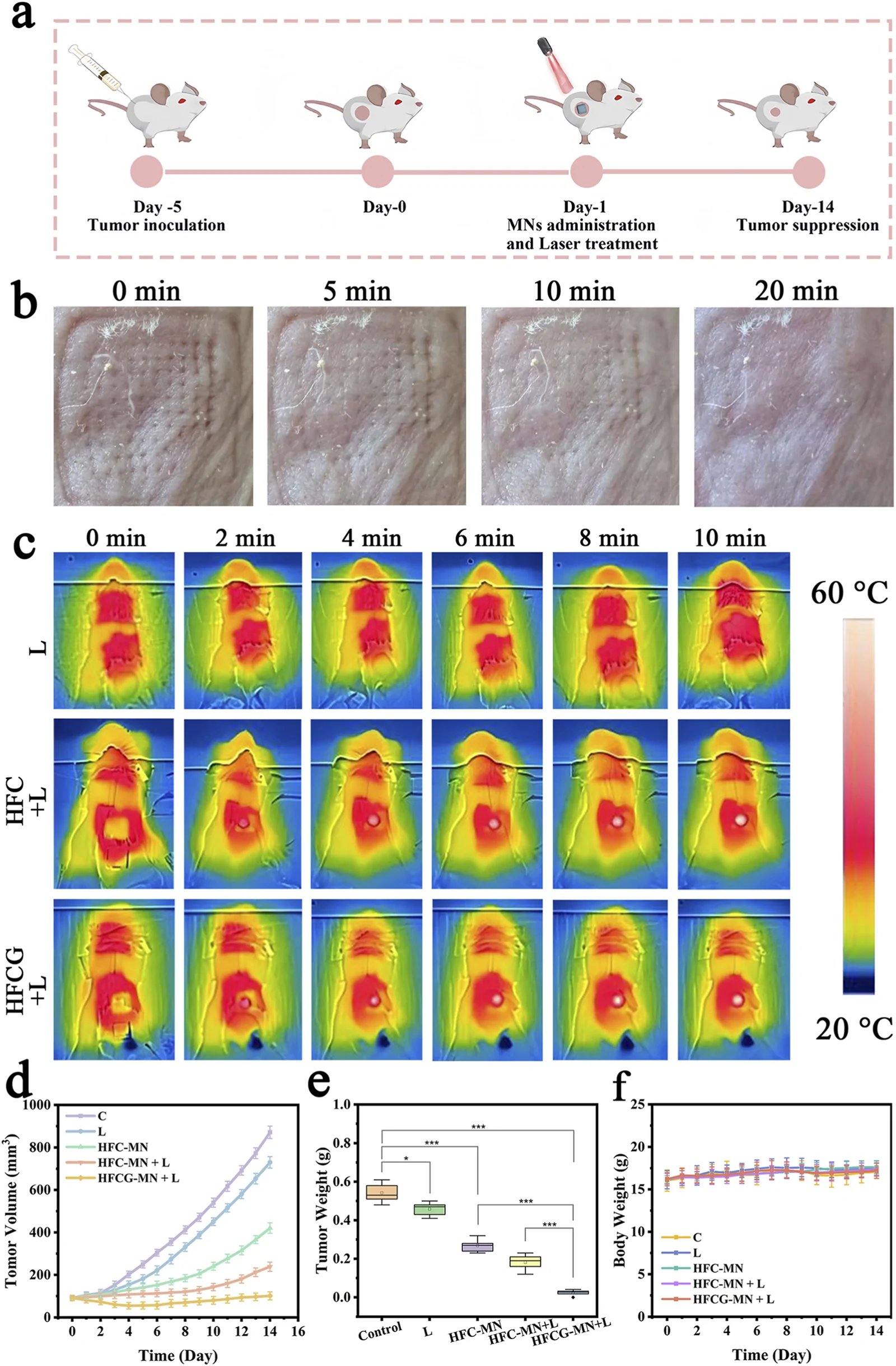
**(a)** Schematic illustration of the treatment schedule for 4T1 tumor-bearing mice. **(b)** Representative photographs of mouse skin at 0, 5, 10, and 20 min after HFCG-MN insertion, showing the gradual closure of microneedle-induced microchannels. **(c)** Infrared thermal images and corresponding temperature changes at the tumor sites of mice under laser irradiation. **(d)** Tumor growth curves of 4T1 tumor-bearing mice after different treatments. **(e)** Average tumor weights of mice in different treatment groups on day 14. **(f)** Body-weight changes of mice during the 14-day treatment period.

Representative photographs of the mice collected on days 0, 3, 7, and 14 further illustrated the therapeutic outcomes (Supplementary Figure S18). Tumors in the control group progressively increased in size, whereas those in the HFCG-MN + L group gradually decreased after treatment. Gross images of the excised tumors further showed that the tumors from the HFCG-MN + L group were substantially smaller than those from the other groups (Supplementary Figure S19), consistent with the tumor growth curves and tumor weight measurements. Together, these results demonstrate the potent *in vivo* antitumor activity of HFCG-MN combined with laser irradiation against 4T1 tumors. Finally, the *in vivo* biosafety of HFCG-MN was evaluated by histological analysis of the major organs. H&E staining showed no obvious pathological abnormalities in the heart, liver, spleen, lung, or kidney of mice from any treatment group, with tissue morphology comparable to that of the control group. In contrast, tumor tissues from the HFCG-MN + L group exhibited extensive cell destruction, necrotic regions, and pronounced loss of tissue integrity compared with the relatively intact tumor morphology observed in the control group (Supplementary Figure S20). These histological findings further support the strong antitumor efficacy of HFCG-MN-mediated photothermal treatment while showing no obvious damage to the major organs under the tested conditions.

## 4 Conclusion

In summary, this study establishes a coherent structure–delivery–mechanism strategy for local tumor therapy. Hollowing and stepwise calcination transformed FeSnO(OH)_5_ into an oxygen-deficient Fe_3_O_4_@SnO_2_ nanozyme with improved light absorption, charge transfer, photothermal stability, and multi-ROS generation. Embedding HFC and GA in a PAAs/PVA/PVP dissolving microneedles preserved these functions while adding mechanical penetration, rapid dissolution, and pH- and light-responsive release. PAAs and PVA formed the supportive matrix, while PVP served as a hydrophilic, rapidly dissolving co-matrix component. At the cellular level, the platform connected ROS generation and GSH depletion to Fe^2+^ accumulation, lipid peroxidation, lysosomal leakage, and mitochondrial depolarization. At the organism level, local HFCG-MN plus laser treatment produced the strongest tumor control with no obvious systemic toxicity in the available readouts. The main conceptual advance is therefore not a simple combination of modalities, but a spatially confined redox cascade in which defect-engineered nanozyme chemistry and microneedle delivery reinforce one another. Ferroptosis-rescue experiments, pathway proteins, pharmacokinetics, and long-term recurrence studies will be important to define the mechanism and translational window more rigorously.

## Data Availability

The original contributions presented in the study are included in the article/Supplementary Material, further inquiries can be directed to the corresponding authors.
