# Supplementary material for: Defect-engineered Fe3O4@SnO2 nanozyme microneedles couple local photothermal-photodynamic therapy with redox-amplified ferroptosis
Source: Front Chem. 2026 Oct 19;14:1991252. doi: 10.3389/fchem.2026.1991252 (PMC13725851; doi:10.3389/fchem.2026.1991252)
Supplement: Supplementary file 1 [file DataSheet1.docx]

Supplementary Material

Defect-Engineered Fe_3_O_4_@SnO_2_ Nanozyme Microneedles Couple Local Photothermal-Photodynamic Therapy with Redox-Amplified Ferroptosis

Wei Guo^1,2*^, Wenbo Wang^2^, Zhuoran Yang^1*^, Liqiang Jing^1*^

^1^Key Laboratory of Functional Inorganic Materials Chemistry (Ministry of Education), School of Chemistry and Materials Science, International Joint Research Center for Catalytic Technology, Heilongjiang University, Harbin 150080, China

^2^Key Laboratory of Photochemical Biomaterials and Energy Storage Materials, Heilongjiang Province and College of Chemistry and Chemical Engineering, Harbin Normal University, Harbin 150025, China

* Correspondence:

guowei@hrbnu.edu.cn

Zhuoran16677@163.com

Jinglq@hlju.edu.cn

FIGURE S1 SEM images of FeSnO(OH)_5_ synthesized using different iron sources: (a) FeSO_4_, (b) FeCl_2_, and (c) (NH_4_)_2_Fe(SO_4_)_2_; and with different amounts of FeCl_2_: (d) 1 mmol, (e) 2 mmol, and (f) 4 mmol.

FIGURE S2 SEM images of HF obtained by etching with NaOH of different concentrations: (a) 6 M; (b) 8 M; (c) 10 M; (d) 12 M.

FIGURE S3 TEM and EDS mapping images of HF.

FIGURE S4 (a) XRD pattern and (b) particle size distribution pattern of HF.

FIGURE S5 SEM images of one-step calcination of HF at different temperatures: (a) 300 °C, (b) 450 °C, (c) 600 °C, and (d) 750 °C.

FIGURE S6 SEM and XRD images of HF calcined in two steps at varying temperatures: (a, e) 600 + 300 °C; (b, f) 600 + 450 °C; (c, g) 600 + 600 °C; (d, h) 450 + 450 °C.

FIGURE S7. Temperature changes of HFC solutions with different laser intensities under 655nm laser irradiation.

FIGURE S8. Time-dependent oxidation of TMB in the presence of FeSnO(OH)_5_, HF, HFC-1, and HFC under 655 nm laser irradiation.

FIGURE S9. TMB oxidation induced by HFC under 655 nm laser irradiation at different power densities (0.5, 1.0, and 2.0 W cm^-2^).

FIGURE S10. Concentration-dependent TMB oxidation by HFC at pH 5.4 and 7.4.

FIGURE S11 (a) GSH Depletion by HFC over time; (b) UV-vis absorption changes during GSH depletion of different samples.

FIGURE S12 (a) Fluidity test and (b) image of microneedles prepared with different amounts of PAAs (0.05, 0.1, 0.2 g).

FIGURE S13 (a) fluidity test, (b) image; (c) tin foil penetration test of different concentrations of PVA (0.5, 1.0, and 1.5 g) added to PAAs (0.1 g) hydrogel.

FIGURE S14 (a) Cross-section images of the MNs loaded with MB inserted into the agar gel; (b) solubility test of HFCG-MN over time.

FIGURE S15 Intracellular Fe^2+^ fluorescence quantitative analysis in 4T1 cells

FIGURE S16 The JC-1 red/green fluorescence images in 4T1 cells subjected to different treatments.

FIGURE S17 Live/dead cell fluorescence quantitative analysis in 4T1 cells.

FIGURE S18 Representative photographs of tumors excised from mice after different treatments on day 14.

FIGURE S19 Representative photographs of mice from different treatment groups recorded on days 0, 3, 7, and 14.

FIGURE S20 H&E staining of major organs (heart, liver, spleen, lung, and kidney) and tumor tissues from mice after different treatments.
